# Robust in-vehicle respiratory rate detection using multimodal signal fusion

**DOI:** 10.1038/s41598-023-47504-y

**Published:** 2023-11-22

**Authors:** Joana M. Warnecke, Joan Lasenby, Thomas M. Deserno

**Affiliations:** 1 Peter L. Reichertz Institute for Medical Informatics of TU Braunschweig and Hannover Medical School, 38106 Braunschweig, Germany; 2 Department of Engineering, University of Cambridge, Cambridge, CB2 1PZ UK

**Keywords:** Biomedical engineering, Respiratory signs and symptoms

## Abstract

Continuous health monitoring in private spaces such as the car is not yet fully exploited to detect diseases in an early stage. Therefore, we develop a redundant health monitoring sensor system and signal fusion approaches to determine the respiratory rate during driving. To recognise the breathing movements, we use a piezoelectric sensor, two accelerometers attached to the seat and the seat belt, and a camera behind the windscreen. We record data from 15 subjects during three driving scenarios (15 min each) city, highway, and countryside. An additional chest belt provides the ground truth. We compare the four convolutional neural network (CNN)-based fusion approaches: early, sensor-based late, signal-based late, and hybrid fusion. We evaluate the performance of fusing for all four signals to determine the portion of driving time and the signal combination. The hybrid algorithm fusing all four signals is most effective in detecting respiratory rates in the city ($P = 62.42$), highway ($P = 62.67$), and countryside ($P = 60.94$). In summary, 60% of the total driving time can be used to measure the respiratory rate. The number of signals used in the multi-signal fusion improves reliability and enables continuous health monitoring in a driving vehicle.

## Introduction

The respiratory rate is one out of four primary vital signs to derive the health status of individuals^1,2^. It is significant to predict adverse cardiac events^3^, emotional stress^4^, and cognitive load^5^. Nicolo et al. defined thirteen goals for respiratory monitoring^1^, ranging from the presence of breathing to cardiac events as well as environmental stress^1^. Moreover, the respiratory rate provides information for airways and other lung structures, which are affected by chronic respiratory diseases (CRDs)^2^. Asthma, chronic obstructive pulmonary disease (COPD), occupational lung disease, and pulmonary hypertension are some of the most prevalent^6^. According to the World Health Organisation (WHO), more than 262 million people suffer from asthma^6^. In most cases, asthma can be treated with inhaled medications^7^. However, COPD is mostly irreversible and particularly affects adults^8^. It is characterized by breathlessness, sputum production, and chronic coughing^6^. Furthermore, COPD patients develop more severe COVID-19 symptoms^9^. Continuous health monitoring and regular medical check-ups yield early detection of symptoms, improve therapeutic outcomes for CRDs, and prevent cardiac events^10^. In Western countries, people spend, on average, 35 min per day in a car^11^. Therefore, unobtrusive in-vehicle sensors can potentially integrate continuous health monitoring into our daily lives^12^.

Currently, in-vehicle monitoring focuses on the tiredness tracking of the driver and uses eye- and face-tracking or steering wheel, pedal, and lane movements^13,14^. More recently, state-of-the-art research also focuses on vital signs recorded in automotive environments, in particular the respiratory rate^15,16^. For instance, Ju et al. integrated a piezoelectric sensor in the seat belt to derive the drivers’ stress level under laboratory conditions^17^. Baeck et al. applied similar sensors while driving^18^. Vavrinsky et al. used ballistocardiography (BCG) in a bucket seat^19^. Vinci et al. suggested radar sensors in the seat backrest at the height of the lunge to measure the respiratory rate^20^. Guo et al.^21^ used a near-infrared camera for respiratory rate detection. However, current research is limited mostly to only one particular sensor concept and aims at supporting driving assistance systems rather than monitoring the long-term health status of vehicle occupants^15^. In previous work, we used an accelerometer attached to the seat belt under different driving conditions to determine the best position^22^ and a de-noising method for respiratory rate monitoring^23^.

In this work, we present a redundant sensor system and signal fusion approaches for in-vehicle respiratory rate detection to answer the following research question: (1) *Which portion of the driving time is utilisable to measure the respiratory rate robustly?*, (2) *Which sensor combination delivers the most reliable results?*, and (3) *What is the performance difference for respiratory rate detection between the driving scenarios?*.

## Material 

### Sensor system

**Figure 1:**
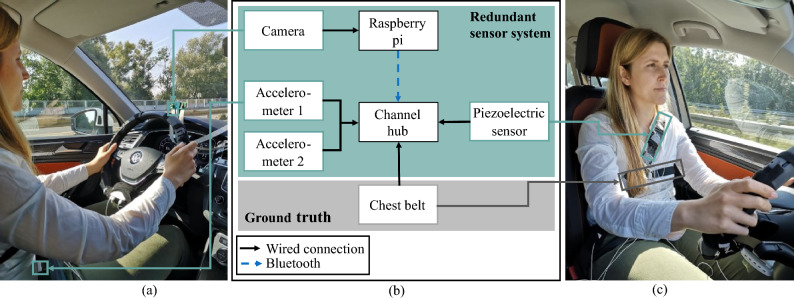
Sensor system. (**a**) The camera is behind the steering wheel, and the accelerometers are in the seat belt as well as on the right side of the driver’s seat. (**b**) Schematic sensor system. (**c**) The piezoelectric sensor sits in the seat belt, and the chest belt for reference measurements is strapped around the subject’s chest.

Based on our previous review^16^ and the performance assessment by Leonhardt et al.^15^, we choose two accelerometer sensors (BiosignalPlux Explorer, Plux Wireless Biosignals, Lisboa, Portugal), a piezoelectric sensor (BiosignalPlux Explorer, Plux Wireless Biosignals, Lisboa, Portugal), and an RGB camera (Raspberry Pi Foundation, Cambridge, United Kingdom). The camera has a resolution of 1280 $\times$ 720 pixels and records with ten frames per second (Fig. 1a). The channel hub (BiosignalPlux Explorer, Plux Wireless Biosignals, Lisboa, Portugal) connects the accelerometer, piezoelectric sensor, and chest belt and sends the recordings via Bluetooth, and the camera has a wired connection to the Raspberry Pi (Fig. 1b).

For ground truth measurements, we use a chest belt (BiosignalPlux Explorer, Plux Wireless Biosignals, Lisboa, Portugal) (Fig. 1c). We place the belt around the upper thorax of the subject and measure the peaks of the movement. According to our previous work^23^, we attach the accelerometers to the seat belt and the seat to measure noisy breathing movements and noise only, respectively (Fig. 1a). We integrate the piezoelectric sensor into the seat belt and attach the camera behind the steering wheel (Fig. 1c). We install all sensors into a street-legal vehicle (VW Tiguan, Volkswagen AG, Wolfsburg, Germany). To determine the respiratory rate optically, we average the green channel for specific regions of interest (ROI): (1) belt and (2) chest.

### Experimental setup

We selected 15 test persons with different ages (20–67 years), genders (female: 6, male: 9), and body mass index (21–44). For each subject, we record 15 min of driving: (1) in the *city*, (2) on the *highway*, and (3) in the *countryside*. Following the navigation system, all subjects drove the same predetermined route.

Existing studies often show the technical feasibility in a simulated environment with a low number of subjects^17,19,20^. Guo et al. conducted one experiment with five subjects under real driving conditions with a near-infrared camera^21^. The advantage of our study is the recording of various sensors for respiratory rate detection, three different driving scenarios, a higher number of subjects, and a publicly available data set.

Driving scenarios, such as highway, city, and countryside, are different due to a variety of factors, including road types, traffic conditions, speed limits, environmental variables, and the types of challenges. Therefore, we recorded data in three scenarios. Different driving scenarios feature distinct road conditions. Highways typically have well-defined lanes and relatively stable traffic patterns, while city roads often involve frequent lane changes, pedestrians, and complex traffic dynamics. The countryside has more uneven roads because the road conditions are often not as good as on the highway or in the city center. Collecting data in all scenarios enables a comparison between these different scenarios to analyse the data recorded with various road conditions. Each scenario has various traffic patterns. The highway typically has high-speed limits, while city driving involves frequent stops and starts and congestion. Countryside includes fewer vehicles and lower speed limits but can pose challenges like winding roads and variable road quality. The speed limit for the highway was 130 km/h, for the city 50 km/h, and for the countryside between 50 and 100 km/h.

### Ethics approval

We record all data anonymously following the Helsinki Declaration. The ethics committee at TU Braunschweig (Braunschweig, Germany) approved the study’s procedures (internal process number: D_2022-13). Informed consent was obtained from all subjects. Specific consent was obtained for identifying images in an online open-access publication.

## Signal pre-processing

### Piezoelectrical sensor

The piezoelectrical sensor determines the pressure that is generated when the breathing chest expands against the seat belt. As the sensor directly turns the pressure into an electrical signal (voltage), we do not apply additional preprocessing but use the raw data directly.

### Accelerometers

**Figure 2:**
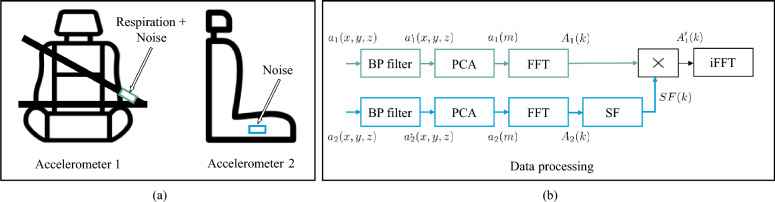
Pre-processing of the accelerometer signals. (**a**) Sensor positions. (**b**) Processing pipeline.

For noise reduction, we attached *accelerometer 1* to the seat belt at the position side-waist, which measures the respiration and the noise (Fig. 2a). We selected the position side waist based on a previous publication^22^, which evaluated the positions of the shoulder, chest, side waist, and waist for respiratory rate detection with an accelerometer. The *accelerometer 2* is attached to the seat of the driver on the right side and measures the environmental noise (Fig. 2a). The required pre-processing of the signals uses bandpass (BP) filtering and principal component analysis (PCA). The noise canceling is then computed in the Fourier domain, using the fast Fourier transform (FFT) (Fig. 2b).

The magnitude in $X_1$ of the *accelerometer 1* includes the frequency distribution of respiration plus noise and $X_2$ just the noise. In $X_2$, a higher magnitude of the frequency component implies higher noise. Specifically, $|X_2(k)|$ denotes the kth amplitude in an FFT series of *accelerometer 2*. Using Eq. (1), we calculated the suppression factor (*SF*) that suppresses the frequency components in $X_1$ based on $X_2$ magnitudes and $\mu (|X_1|)$ is the mean value of the $X_1$ amplitudes^23^:

1 $$\begin{aligned} SF(k)= e^{-\frac{|X_2(k)|}{(\mu (|X_1|)}} \end{aligned}$$

Using the suppression factor (SF) in Eq. (2), we calculated the frequency distribution of the suppressed signal (supp):

2 $$\begin{aligned} X_{supp}(k)= SF(k) \cdot {X_1(k)} \end{aligned}$$

The output is a de-noised signal for sensor $A{'}_1(k)$. In contrast to the previous publication, we applied the inverse FFT (iFFT) because the signal fusion approach needs the signal in the time domain as an input^23^. For further details, we refer to our previous work^23^.

### Video

**Figure 3:**
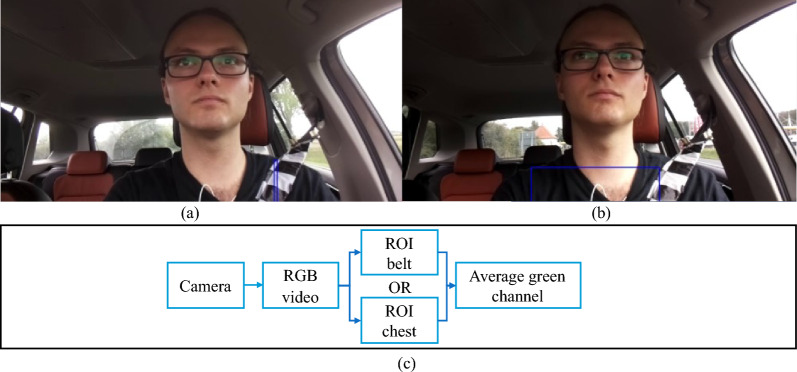
Video signal. (**a**) ROI belt. (**b**) ROI chest. (**c**) Data processing.

We attached a black-and-white chessboard pattern on the seat belt to increase the contrast between the clothes and the seat belt. This makes our vision system unresponsive to the color of the clothing the subject is wearing. Using the static setting in the car, we extract two ROIs covering the belt ($video_{Belt}$) and chest ($video_{Chest}$) (Fig. 3). The ROIs are a rectangle and determined by the static positions of the camera as well as the driver’s seat: $video_{Belt}$ (x: 701, y: 550, w: 10, h: 190) (Fig. 3a) and $video_{Chest}$ (x: 401, y: 550, w: 400, h: 190) (Fig. 3b).

This means that the ROI belt has a width of 10 pixels and a height of 190 pixels at positions x: 701 and y: 550. To ensure the accuracy of ROI extraction, we verify their positions during the pre-processing stage and make necessary position adjustments. Using Eq. (3), The average (avg) green colour values of each cut-out pixel in the ROI are calculated by adding up all the color values and dividing by the total number of values:

3 $$\begin{aligned} \text {color}_{\text{ avg }}=\frac{1}{w\cdot h} \sum _{w=1}^{n} \sum _{h=1}^{m} \text{ Pixel}_{w,h} \end{aligned}$$

The average colour values of the ROIs change with every in- and exhaling movement because the position of the belt changes. Therefore, the respiratory rate can be calculated based on the movement of the belt.

### Implementation for statistical analysis

To derive the signal from the ROI of $video_{Belt}$ and $video_{Chest}$, we use the library OpenCV (version 4.5). We implement the CNN-based approach in Python (version 3.8.5) using the libraries TensorFlow (version 2.3.1) and Keras (version 2.4.3). The high-performance computer cluster *Phoenix* calculates all training and testing^24^. For the evaluation, we use the library Numpy (version 1.19.0).

### Input data

**Figure 4:**
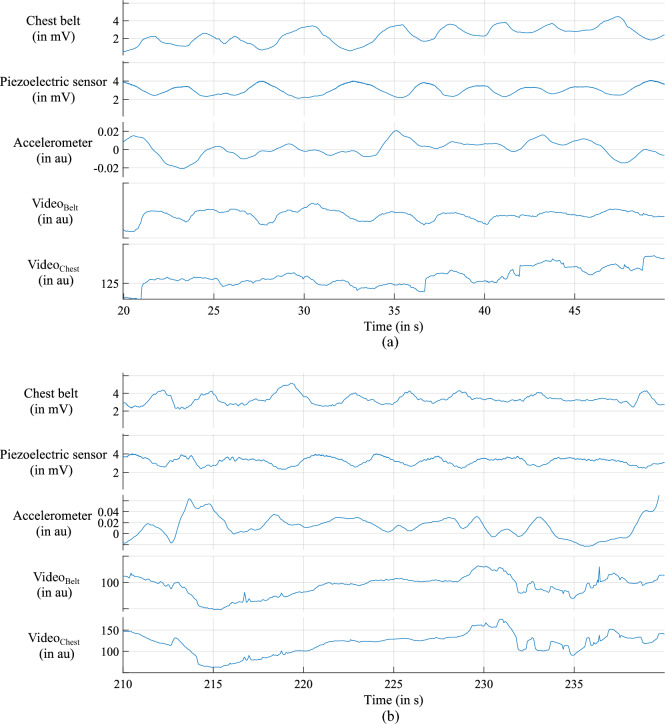
Input data from subject 5678 during the scenario *city* (**a**) Input data with low SNR from 20 to 50 s. (**b**) Input data with high SNR from second 210 to 240. We created the visualisation with MATLAB (MATLAB version R2021a, The MathWorks, Natick, United States).

In total, our sensors deliver four input channels and one reference signal as ground truth. The recordings differ in signal-to-noise ratio (SNR). Figure 4 gives examples of lower (Fig. 4a) and higher SNRs (Fig. 4b), respectively. The arbitrary unit (au) represents the unit for the accelerometer and the video signal.

According to Chandra et al. ^25^, we apply additional pre-processing for signal fusion:

1. Upsampling all data to a unified sampling rate of 200 Hz,
2. Median filtering, and 3. Amplitude normalization to the interval $[-1,1]$.

We split the signal into snippets of 201 overlapping samples. Following Chandra et al.^25^, test and training snippets overlap by 200 and 190 data points, respectively. We use the leave-one-subject-out cross-validation scheme: we train the CNN on 14 subjects and use the data of the remaining subjects for testing. We repeat this procedure 15 times and average the results.

## Signal fusion

**Figure 5:**
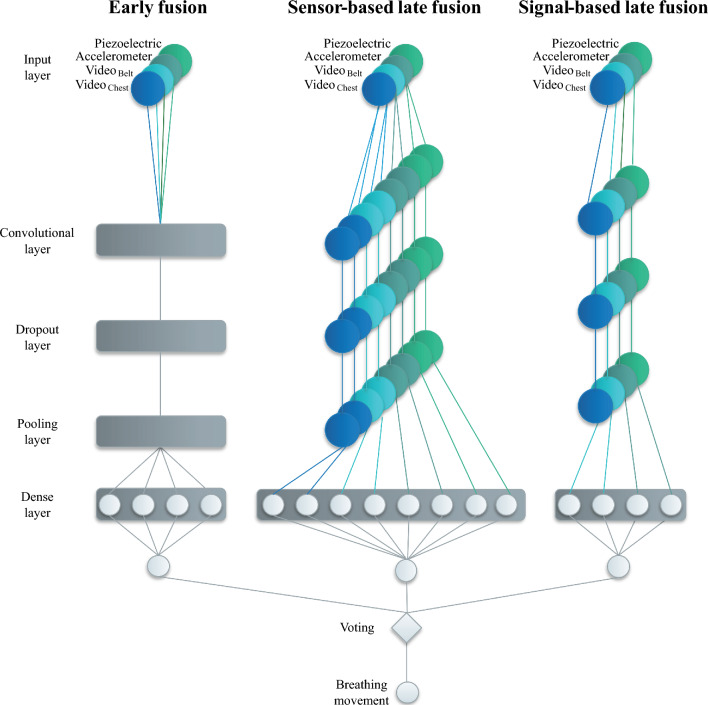
Hybrid signal fusion for multiple sensors.

We calculate the performance of (1) early fusion, (2) signal-based late fusion, (3) sensor-based late fusion, and (4) hybrid fusion (Fig. 5). The latter combines the other three with a majority voting^26^. Many authors reported that one sensor is insufficient to measure vital signs in a medical setting and suggested redundancy with respect to both the number of sensors and the physical base of sensor systems^15,16^. However, signals from multiple sensors need a fusion strategy. The algorithm from Chandra et al. estimates the heartbeat location of multiple signals based on a convolutional neural network (CNN)^25^. This general approach can be applied to other data. Münzner et al. compared early fusion with several late fusions^27^.

Early fusion merges in a first convolutional layer. Sensor-based late fusion merges in the dense layer, and signal-based late fusion with an increased number of extracted features. For respiratory rate detection, we combine these approaches and extend them with a majority vote.

The first layer receives our five input signals. The second layer is a convolutional layer, which generates a feature map. To extract the features, we use four filters with a length of 20 samples for each of the sensor signals^25^. To prevent over-fitting, we use a dropout rate of 0.5 in the dropout layer. The pooling layer reduces the dimension of the feature map. The dense layer classifies snippets into binary classes 1 and 0, which means that the snippet contains a heartbeat or not, respectively. The activation function is a sigmoid function. The output layer generates a vector *Y* of multiple labels that are either 0 or 1 for a specific snippet. The majority vote takes place after the output layer.

An early fusion approach uses a single CNN and fuses signals within the convolutional layer (Fig. 5). By using two CNNs per signal, the sensor-based late fusion method extracts more features per signal. The signal-based late fusion uses one CNN per signal. Both of these approaches merge in the dense layer.

## Evaluation

Comparing snippets with ground truth, we obtain true-positive (TP), true-negative (TN), false-positive (FP), and false-negative (FN) classifications. We calculate performance $P = (PPV+S)/2$ based on the positive predictive value $PPV = TP/(TP+FP)$ and the sensitivity $S = TP/(TP+FN)$, as these evaluation metrics reflect the FP and FN numbers^25^.

We first evaluate the different sensors and their combinations, disregarding the fusion algorithms and the driving scenarios. We then determine the best type of fusion and analyse the impact of the different driving scenarios. Finally, we determine the portion of driving time that can be used for reliable respiratory rate measurements.

## Results

### Comparison between one signal

The tables compare the average performance during the three driving scenarios for the four sensors to identify the signals with the best performance (Table 1). The signal-based late fusion yields the highest performance ($P_{max}=55.79$) with the signal accelerometer (Table 1). In summary, the video$_{Belt}$ has the highest score twice, and the accelerometer and video$_{Chest}$ have the highest score once for a signal fusion approach. We will describe the de-noised accelerometer signal as *Acc* and the piezoelectric signal as *Piezo* in the following text to simplify the notation.

**Table 1:** Performance in percent for one signal during the three driving scenarios.

Approach	Piezo [%]	Acc [%]	Video$_{Belt}$ [%]	Video$_{Chest}$ [%]
Early fusion	49.08	49.16	49.66	**53.38**
Signal-based late fusion	49.10	**55.79**	51.80	49.51
Sensor-based late fusion	49.10	49.19	**53.58**	51.40
Hybrid fusion	48.82	49.17	**53.31**	52.21

Significant values are in bold.

### Comparison between two signals

The comparison of two sensor pairs shows that signal-based late fusion has the highest performance ($P_{max}=56.08$) with the pair piezoelectric sensor and video$_{Belt}$ (Table 2). The sensor pair video$_{Belt}$ and video$_{Chest}$ has the lowest performance for the early fusion approach ($P=541.21$). In summary, the combination of accelerometer and video$_{Chest}$ achieves the best performance twice, and the piezoelectric and video$_{Belt}$ once.

**Table 2:** Performance in percent for two signals during the driving scenario.

Approach	Piezo+ Acc [%]	Piezo+ Video$_{Belt}$ [%]	Piezo+ Video$_{Chest}$ [%]	Acc+ Video$_{Belt}$ [%]	Acc+ Video$_{Chest}$ [%]	Video$_{Belt}$+ Video$_{Chest}$ [%]
Early fusion	52.35	46.58	**54.25**	50.32	46.58	42.21
Signal-based late fusion	53.18	**56.08**	47.36	48.44	50.89	52.08
Sensor-based late fusion	48.77	48.87	50.63	47.93	**55.15**	46.73
Hybrid fusion	50.32	50.91	50.71	50.13	**51.29**	46.89

Significant values are in bold.

### Comparison between three signals

The three combined signals, piezoelectric signal, accelerometer, and video$_{Belt}$ have the highest score ($P_{max}=55.64$) with the approach signal-based late fusion (Table 3). On the other hand, this fusion approach also has the worst performance with the combination of piezoelectric sensor, video$_{Belt}$, and video$_{Chest}$ ($P=46.29$). However, the combination of a piezoelectric sensor, accelerometer, and video$_{Belt}$ as well as a piezoelectric sensor, video$_{Belt}$, and video$_{Chest}$ yields two times the highest performance per signal fusion approach.

**Table 3:** Performance in percent for three signals during the scenario *city*.

Approach	Piezo+Acc+Video$_{Belt}$ [%]	Piezo+ Acc+ Video$_{Chest}$ [%]	Piezo+ Video$_{Belt}$+ Video$_{Chest}$ [%]	Acc+ Video$_{Belt}$+ Video$_{Chest}$ [%]
Early fusion	52.39	50.83	**53.63**	53.59
Signal-based late fusion	**55.64**	54.36	48.34	46.29
Sensor-based late fusion	48.49	46.96	**51.24**	50.48
Hybrid fusion	**52.35**	49.51	50.67	49.06

Significant values are in bold.

### Comparison between four signals

**Figure 6:**
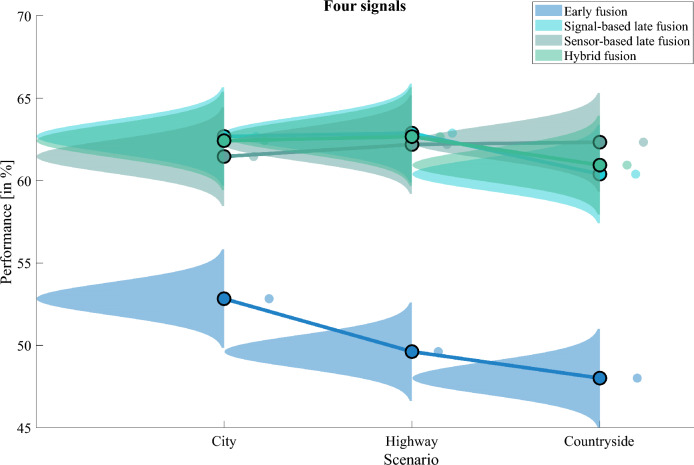
Performance of four signals for different driving scenarios. This plot was created with the MATLAB package from Allen et al.^28^ (MATLAB version R2021a, The MathWorks, Natick, United States).

The raincloud plot visualises the distribution of the performance during the driving scenarios to show the performance differences (Fig. 6). The early fusion approach consistently performs poorly, with the lowest scores in all scenarios and the lowest average performance overall (Fig. 6). However, the distribution for the other fusion algorithms is comparable (Fig. 6).

With the combination of all four signals, the signal-based late fusion approach achieves the highest score ($P_{max}=62.88$) in the scenario *highway* (Table 4). However, the hybrid fusion achieves the highest average performance ($Mean_{P}=62.01$) overall driving scenarios. The early fusion technique yields the lowest average performance ($Mean_{P}=50.15$).

**Table 4:** Performance in percent for four signals during driving.

Approach	City [%]	Highway [%]	Countryside [%]	Mean$_P$ [%]
Early fusion	**52.83**	49.62	48.01	50.15
Signal-based late fusion	62.68	**62.88**	60.39	61.98
Sensor-based late fusion	61.46	62.19	**62.33**	61.99
Hybrid fusion	62.42	**62.67**	60.94	**62.01**

Significant values are in bold.

## Discussion

The major challenge for health monitoring during driving is the changing data quality due to artifacts, which are caused by movements of the car or driver due to driving activities or talking^15,16^. Moreover, the different physical attributes, e.g., height and weight, of the test subjects lead to a high standard deviation in performance^15^. The iPPG signal is especially sensitive to light changes and movements^29^. Moreover, other methods, such as PCA and FFT, could lead to better signal extraction. Furthermore, it is possible to integrate more sensors into the redundant sensor system. The system could be extended by a magnetic induction sensor integrated into the seat backrest^30^. We excluded the BCG sensor because the pretest in the car showed that the SNR was low. For the pretest, we placed the sensor at the backrest to measure ballistic forces generated by the lungs. We also excluded a radar sensor because the API did not allow sufficient time synchronization. For our pretest, we used the radar sensor from Acconeer A111. The Raspberry Pi and camera are low-cost devices. The reliability of the Raspberry Pi is proven in other publications^31,32^. There is also the possibility of optimizing the structure of signal fusion models. The performance of models with adjusted parameters could be investigated in further studies, e.g., the number of convolutional layers^33^, decreasing or varying the learning rate of the Adam optimizer^34^, or other activation functions^35^. A long short-term memory (LSTM) or autoencoder-based structure could also increase the performance^36^.

The early fusion has the lowest performance with $Mean_{P}=50.15$. This is attributed to the extraction of fewer features compared to sensor-based fusion, which achieves the highest score after the hybrid fusion process. In sensor-based fusion, a more extensive feature set is obtained by employing two CNNs to extract features in the convolutional layer for a single signal, resulting in enhanced performance.

Moreover, it is also important to compare the results from this paper with the literature. Ju et al.^17^ showed the technical feasibility of the pressure sensor integrated into the seatbelt, and Vavrinsky et al.^19^ of a pressure sensor integrated into the seat to measure the respiratory rate. However, they did not collect data from various subjects and presented an evaluation. Beak et al. measured the electrocardiogram (ECG), galvanic skin response (GSR), and respiration to detect the driver’s stress in a study with four male subjects under real driving conditions^18^. For the respiration rate measuring, they integrated a piezoelectric sensor into the seat belt. They did not use a reference sensor such as a chest belt to collect a ground truth for the respiratory rate to evaluate the respiratory rate monitoring because the focus of this study was on the detection of the driver’s stress level. Vinci et al. used a microwave interferometer radar and conducted three measurements for 30 seconds to show the technical feasibility and waveform of the signal in a simulated environment^20^. Guo et al. recorded data with a near-infrared time-of-flight camera to derive the respiratory rate of five subjects under real driving conditions and compared the results with the measurements of a chest belt as a reference^21^. The authors calculated the RR per minute. In contrast to our evaluation, they did not compare the position of the breathing movement with the reference. For 43 % of the driving time on the highway, they had a difference of 0 to 3 breaths per minute (BPM) compared to the reference^21^. The novelty of our work lies in the fusion of the different signals for respiratory rate detection. Moreover, we collected data from 15 subjects under three different driving scenarios to enable further algorithm development with these data. The accuracy of the sensor system and fusion with respect to the other evaluation metrics and recordings is not directly comparable.

For future work, we will record the controller area network (CAN) Bus data to detect artifacts that are caused by environmental disturbances. As suggested by Fu et al.^37^, we will also integrate movement detection using depth cameras. Other important vital signs are the heart rate and temperature. Future systems should record all primary vital signs, i.e., body temperature, pulse rate, and blood pressure. Additionally, we will publish a paper for in-vehicle heartbeat detection. The heartbeat is another important vital sign to detect cardiovascular diseases, such as stroke. The study encompasses 19 healthy subjects and adheres to the same experimental design as described in this paper^38^. We publish two papers because the description of the different sensor systems, pre-processing, and analysis would exceed the scope of one journal paper.

## Conclusion

To detect respiratory rate while driving, we developed a redundant sensor system and signal fusion approaches. Based on our results, the hybrid fusion and all four sensors have the highest performance for in-vehicle respiratory rate detection: *city* ($P = 62.42$), *highway* ($P = 62.67$), and *countryside* ($P = 60.94$). The result also shows that the fusion of multiple signals improves robustness. Furthermore, the voting system of the hybrid algorithm not only outperforms the other fusion algorithms but also presents several distinct advantages. This approach to algorithm fusion leverages the strengths of different techniques and combines them in a way that maximizes overall performance. By allowing each component algorithm to contribute its information through a voting mechanism, the hybrid algorithm achieves a higher accuracy and robustness. As a take-home message and to answer the initial research questions: *We can monitor the respiratory rate continuously for over 60 percent of our driving time with a low variance between the driving scenarios, and the combination of all sensors delivers the most reliable performance*. In summary, the results show the potential to detect CRD symptoms in an early stage.

## Data Availability

We publish the anonymous data in text (.txt) file format over the data storage system of TU Braunschweig under CC BY 4.0 to allow researchers to reproduce and apply further algorithms (link: https://doi.org/10.24355/dbbs.084-202210201440-0, accessed on November 16th, 2023): chest belt (reference signal), accelerometer 1 and 2, piezoelectric signal, video$_{Belt}$, and video$_{Chest}$, and subject information (subject ID, age, height, weight, gender, and known diseases).
